# What Can Public Health Administration Learn from the Decision-Making Processes during COVID-19?

**DOI:** 10.3390/ijerph21010004

**Published:** 2023-12-20

**Authors:** Andrew Joyce, Emma Risely, Celia Green, Gemma Carey, Fiona Buick

**Affiliations:** 1 Centre for Social Impact, Swinburne University of Technology, Hawthorn 3122, Australia; eriseley@swin.edu.au; 2 Centre for Social Impact, University of New South Wales, Sydney 2052, Australia; celia.green@unsw.edu.au (C.G.); gemma.carey@unsw.edu.au (G.C.); 3 School of Business, University of New South Wales, Canberra 2612, Australia; f.buick@adfa.edu.au

**Keywords:** decision-making processes, COVID-19, public health, administration, organisational capacity

## Abstract

Human decision-making is prone to biases and the use of heuristics that can result in making logical errors and erroneous causal connections, which were evident during COVID-19 policy developments and potentially contributed to the inadequate and costly responses to COVID-19. There are decision-making frameworks and tools that can improve organisational decision-making. It is currently unknown as to what extent public health administrations have been using these structured organisational-level decision-making processes to counter decision-making biases. Current reviews of COVID-19 policies could examine not just the content of policy decisions but also how decisions were made. We recommend that understanding whether these decision-making processes have been used in public health administration is key to policy reform and learning from the COVID-19 pandemic. This is a research and practice gap that has significant implications for a wide range of public health policy areas and potentially could have made a profound difference in COVID-19-related policy responses.

## 1. Introduction

The impact of COVID-19 is considered a key reflection point for public administration research and practice, and one of the issues considered of paramount importance is reform to decision-making governance and processes, particularly with respect to addressing decision-making biases [1]. It has been contended that human cognitive biases have shaped poor and inadequate policy responses to COVID-19 in numerous jurisdictions [2]. One of the key biases uncovered was the misperception of case growth as linear, rather than exponential, growth [3]. This was of significance because it had resulted in politicians and the public underestimating the threat of COVID-19 based on low early numbers and hindered efforts towards creating effective early responses [3].

Cognitive biases, selective use of evidence, aligning with research consistent with prevailing political values, and advisory committees comprised of experts all from one discipline type rather than interdisciplinary teams, all contributed to aerosol spread being dismissed, droplet theory promoted, and a whole host of policy responses that proved drastically inadequate [4]. It was concluded that what was needed were interdisciplinary approaches for advisory committees and better decision-making governance structures and processes that make use of a range of expertise [4]. This was viewed as an issue in relation to one of the key vaccine decision-making authorities in Australia, where the selective composition of this committee was noted as a barrier to effective decision-making [5].

A number of biases have been used to explain the priority given to expensive and unnecessary resources that went into purchasing ventilators for intensive care units, relative to early efforts in prevention initiatives, such as physical distancing, testing, and contact tracing, which would have resulted in more lives saved [6]. These policy responses occurred as a result of the following biases: optimism bias, which meant policies were guided by best-case rather than worse-case scenarios; omission bias, which was the preference to fail through inaction rather than through a deliberative action; and prioritising visible (i.e., a sick person) over statistical information on lives saved from prevention measures. The ways to counter these biases were presented around narrative frames and specific legal requirements on estimating lives saved or life-years gained by specific policies [6]. It has also been outlined how the use of emotional and cognitive regulation decision-making models could have improved COVID-19 policy-based resource decisions in the U.K. [7]. Adequately addressing cognitive biases required organisational decision-making processes and rules that could mitigate them [8,9], which also included emotional regulation at the individual and group levels [10]. All these examples illustrate how decision-making processes to address the cognitive biases surrounding COVID-19 were potentially lacking in public health administration jurisdictions in Australia and in many other countries.

There are some investigations currently underway that could examine how to improve the decision-making processes of public health administration bodies. The U.K. Government announced its COVID inquiry in 2022, and the Australian Government also commenced an inquiry into its response to COVID-19 for the purpose of improving responses in future pandemics [11,12]. The U.K. inquiry included a reference for examining “how decisions were made” with suggestions by researchers put forward on how the decision-making process could have been improved with respect to the use of scientific evidence [1]. The extent to which the use of evidenced-based models for decision-making from psychological science and management science has been reviewed in the U.K. inquiry was not clear. For the Australian inquiry, there has been no mention of examining the process of decision-making, and the terms of reference have been largely focused on content issues related to the specific policies, programs, and measures required for an effective pandemic response. Understanding and improving on the decision-making process is also fundamental. COVID-19 required dynamic decision-making, which would be also expected in future pandemics, and it is essential to understand how to improve the quality of the decision-making process [7]. Opportunities to examine the decision-making processes of elite decision-makers in government are rare, and inquiries could substantially improve the decision-making processes of public health administration bodies.

This review firstly illustrated how public health administrations have applied the research on decision-making biases in public campaigns and programs to influence health behaviours, such as through the use of nudge theory. This is an example of applying the research at an individual level. There was a noticeable gap in the research on decision-making biases and how to improve decision-making processes at the administration level itself. This omission of best-practice decision-making processes was very costly during COVID-19. In contrast, other professions have decision-making processes at an organisational (meso) level to counter human decision-making biases. In this paper, we highlight how different industries have implemented approaches to improve decision-making at an organisational (meso) level. This is in contrast to public health, which has tended to focus on an individual level of decision-making. We argued there was both a practice and a research gap around decision-making processes at an organisational level in public health administrations. Lastly, the paper concludes with the recommendations for current policy reviews and future research.

## 2. Application of Decision-Making Research in Public Health

Over the last few decades, there has been considerable research demonstrating that human decision-making deviates from logical expectations in quite consistent patterns [9]. Some of the common cognitive biases as described by Kahneman are that emotional intensity is stronger for loss aversion than potential gain and this combined with over weighting of improbable outcomes means people in desperate situations will take higher risks [9]. Rare events will be overweighted dependent on the ease with which they come to mind, the confirmation bias. Certain features of the information will exacerbate this tendency such as explicit description and concrete representations. As described at the start of this paper, there have been a number of decision-making biases evident during COVID-19 policy responses, such as confirmation bias and optimism bias.

An acknowledged limitation of the behavioural models of decision-making has been their historic neglect of the role of emotion [13]. A review analysed this burgeoning area of research, which examines how emotions influence decisions [13]. There are a number of ways in which this occurs. Firstly, there are the direct emotions associated with the decision itself, for example feeling anxious about a financial choice. Then there is the role of emotions indirectly influencing decisions where research has revealed that emotions not linked with the actual decision, such as feeling happier due to sunny weather or how well your football team is faring, can influence unrelated decisions. The strength of emotion and also the type of emotion can influence decision making processes with different emotions eliciting different types of biases. For example, anger and sadness will elicit different types of thought patterns related to risk and appraisal of other situations. Emotions can also influence the depth with which information is analysed with anger and happiness associated with less deliberate thought and greater reliance on heuristics, relative to sadness. However, negative emotional states can also lead to rumination with one study demonstrating an increased propensity for the anchoring bias. Emotions can influence goal attainment and thus the types of decisions that are made, and interpersonal decisions.

There has also been a large body of research on the social processes influencing decisions [14]. Groups can enhance decision making due to error reduction whereby multiple views reduce the error margin from an individual decision and through the aggregation of knowledge. The composition of the team and processes involved in arriving at the decision influence how well this knowledge is used. Having a diversity of experience and knowledge can enhance the decision depending on processes used. The occurrence of common knowledge to be discussed more than knowledge unique to individual members, relationship tension, perceptions of trust and safety, domineering leadership, and cultural factors related to collectivism can all influence use of divergent knowledge. There is also the tendency for group members to think more alike after working together previously and considerations of bringing new group members versus the cost to group cohesion and trust need to be factored.

Social influence (both normative and informational influences) and intergroup stereotypes (e.g., group differences in age, gender, race, etc.) can also influence group dynamics and the decision-making process [14]. People also value their own opinion more so than others and those of people that are similar to them. This can also constrain the possible benefits from a group decision making process. Thus there are a series of recommendations that managers can follow to enhance decision making processes such as letting people think about the problem individually before doing so as a group; ensuring there is cognitive diversity and that demographic diversity is not being used as its proxy; putting in place processes to encourage debate and trust; balancing team composition to bring in new perspectives while still retaining group cohesion; and being aware of group differences and identities and working to create connection across diverse identities to overcome the “us versus them” conflict. The research on social processes and decision-making has been largely applied in organisational business settings.

In fields such as medicine, engineering, and business, there have been numerous research-based papers and practice guides on how organisations could implement processes based on cognitive science research in order to improve decision-making quality to mitigate cognitive biases [8,9,15,16,17,18,19]. In the field of strategic management, models and frameworks have been developed that integrate cognitive and emotional regulation at an organisational level to improve decision-making [7,10]. This has recently been applied in an illustrative sense to COVID-19 health care policy management in the U.K. [7].

The public health administration field has utilised research on cognitive and decision-making biases, specifically focused on behaviour-change programs for the public. One of the most common ways in which this research has been applied was through the use of nudge theory, which guided behaviour-change initiatives. Nudge-theory-inspired programs have been used by both U.K. and U.S. administrations related to policy initiatives designed to influence behaviour, such as opt-out systems for pension plans, and have been studied with respect to vaccination programs [9,20]. A systematic review of interventions based on nudge theory revealed that shifting individual health choices, particularly dietary behaviour, was the most common application [21]. The field of sustainability was the next most common field in which it has been employed. Particular tactics used in these interventions included altering choice defaults, such as opt-out-type decision processes, providing a social reference point by giving information on the behaviour of other people, altering the presentation style of the information, and the use of reminders. It was commented in the article that the present quality of nudge interventions was limited by a lack of focus on causal mechanisms linking the intervention technique with the outcomes and the lack of nomenclature around intervention types that hindered the meta-analysis of the field and being able to replicate studies.

Nudge theory has also been used to understand how different government policies and communication strategies could influence public compliance with COVID-related prevention activities of the public, such as social distancing and contact location and tracing [22].

Nudge theory is based on the research of heuristics to alter the context and “choice architecture” involved in shifting behaviour. It has been contended that the focus of governments on nudge theory has deflected attention away from more transformative policies required to address the underlying causes of social problems. Curchin summarised research that showed how cognitive biases were exacerbated under the type of stress experienced in poverty, particularly scarcity, and that these decision-making traits were replicated in those from middle-class backgrounds in experimental research [23]. The policy approach of threatening personal benefits if non-compliant may increase stress and render someone more likely to make seemingly irrational decisions. Thus, Curchin recommended a broader examination of how behaviour is shaped by social structure, rather than the more limited application of nudge theory that examines the immediate context, for more specific types of behaviours [23]. Another major gap in the behavioural public administration research has been the lack of focus on mitigating the decision-making biases of policy-makers themselves [24]. It has been rare to find studies on how to address the cognitive biases of public administrators and politicians [24], and the research that was found lacked a meso- or macro-focus and did not examine how it could be applied to governmental structures [25].

Unfortunately, there has been very little empirical research examining whether politicians exhibited decision-making biases, as with more decision-making experience and enhanced access to information relative to the members of the general public, they may potentially be less prone to these biases [26]. Often, this has just been assumed, and the implications of cognitive biases on policy and political decision-making have been explored only hypothetically with respect to international conflicts [27]. Drawing on examples from the Israeli/Palestinian crises and other key historical events, the authors explored how well researched biases, including positive illusions, fundamental attribution error, illusion of transparency, and loss aversion, might cause protagonists to act in a more aggressive and suspicious or “hawkish” manner than would be warranted with a more objective perspective [27]. For example, optimism bias and the illusion of control could inflate opinions about the probability of winning any conflict, and loss aversion could increase the likelihood of prolonging a conflict that was proving unsuccessful [27]. It was acknowledged that due to the complexity of international-conflict-related decisions, the degree to which cognitive biases influenced the decision-making process could not be ascertained by standard explanations and predictions [27]. However, it was still surmised that the process of sense making familiar to political science could offer some utility in understanding how these biases might have impacted past decisions and those into the future [27].

A study with local government politicians found they were susceptible to the representative heuristic which is one of the biases known to affect probability assessments, in this case basing judgments on existing mental prototypes we have [26]. Another study involving public service personnel in the U.K. showed that a “consider-the-opposite strategy” was effective in attenuating anchoring bias to some degree [28]. Attempts at using nudge theory approaches to change individual bureaucratic decisions around disability insurance payments and employment for clients were not successful [29]. Thus, at present, there has been limited research into addressing cognitive biases of public administrators [24], and there has been a lack of an organisational meso-level focus [25]. There has been no research, of which we are aware, that has trialled and evaluated the use of meso-level organisational decision-making processes within public health administrations to reduce cognitive biases. This was a peculiar gap as while it is very difficult for individuals themselves to correct their own biases, organisations do have the capacity to put in place decision-making processes in order to correct these biases, and this has been well utilised in corporate environments [9].

Another way in which cognitive science research has been applied in political science has been concerning how the public viewed certain policies and politicians [30,31] and how politicians chose certain emotional frames in their communication, which was commented upon in a paper on political responses to COVID-19 [32]. Again, however, this research lacked any coverage of how cognitive biases and emotions influenced the decision-making of elite policy actors and the potential mitigation strategies that could be used to address these biases, such as those recommended for corporations [33]. There seemed to be little research or evidence on how public health administrative organisations themselves could improve their decision-making processes. However, there have been many examples from other industries where this research was applied at the organisational level.

## 3. Improving Decision-Making at an Organisational Capacity Level

The research, to date, on mitigating cognitive biases in public health administration has been conducted at the individual level. There seemed to be a gap in practice at the organisational and policy levels. However, there have been a number of research strands that could support improved decision-making processes in public health administration. Firstly, the concepts from the field of policy capacity could help articulate the gap in public health administration practices. Good decision-making processes have been considered fundamental components of policy capacity [34,35,36]. Indeed, the aim is that the formation of policy is guided by good decision-making characteristics [34]. A popular taxonomy for policy capacity examines system capacity, organisational capacity, and individual capacity [36]. Policy capacity can be construed as a function of the individual skills of the policy makers and the organisational and systems capacity to provide good quality information and monitoring and evaluation functions [35]. Important factors for policy capacity also include strong and clear consultation strategies and defined rules and processes for making decisions [35]. The policy-capacity tool developed by Lawrence et al. had a criteria point of having a decision-making rule or process in place [35]. However, it made no mention of what that rule should be and no coverage of the literature on mitigating cognitive biases. Based on this policy-capacity taxonomy, it should be clear from the previous section that public health administrations have lacked organisational and systems capacities for applying good decision-making processes. There have been models and framework from other disciplines that could be used in public health administration.

Parkhurst developed a framework for understanding cognitive biases from a systems capacity level, involving a political science and cognitive science perspective [37]. In this conceptual framework, Parkhurst detailed how the degree to which the policy problem was complex, contested, and polarising, and whether the potential source of the bias was technical or issues based could help clarify the particular cognitive bias that may be present [37]. This model was at the systems capacity level [36]. In fields such as medicine, engineering, and business, there have been numerous research-based papers and practice guides on decision-making processes at the organisational level that have drawn on cognitive science, with the aim to mitigate cognitive biases and, thus, improve decision-making quality [8,9]. Addressing cognitive biases has been applied extensively in corporate environments at board and managerial levels, with various decision-making-structure recommendations aimed at bias mitigation [33]. There have been a number of organisational decision-making frameworks and models that have combined cognitive and social psychology research with strategic management research [15,38,39].

Entrepreneurs are particularly prone to confirmation bias whereby they seek information that confirms existing beliefs [16], and this bias was also present during COVID-19 decision-making [40]. For entrepreneurs, this is potentially due to the time pressures of starting a business together with their optimistic thinking, which itself is a cognitive bias that renders an increased risk for confirmation bias while discounting other risks as well as other important business considerations [16]. One remedy suggested was to have a list of question prompts on which to reflect on whether cognitive biases may be present in business planning and thinking [9,16]. Another technique is to use the pre-mortem technique developed by Gary Klein [18]. This process involves imagining that the business or project has failed and then coming up with a list of factors to explain this failure. Using a process of hindsight thinking, even if imaginary, could change the nature of the way risks are assessed and is a successful technique for mitigating confirmation bias and optimistic bias [16,18]. In the private-sector-management field, there have been many techniques used for employing decision-making processes in order to minimise individual and social biases, and these have covered how many people to include, what roles they should have in the decision-making process, and how to identify particular decision tasks that each member should fulfil [8]. One important consideration in the application of these cognitive models is understanding what emotions that they may trigger as regulating emotions at individual and group levels is also critical for good decision-making [10].

There were some examples in the public administration of mitigating cognitive biases but none, to the best of our knowledge, that were used in COVID-19 policy decision-making. In public administration research, there has been some focus on managerial biases and gender equity in the public sector, along with proposed approaches to mitigating these biases [41,42,43], and some attempt at addressing racial biases, although such efforts were underwhelming relative to the historical context of racial bias in American public administrations [44]. The World Bank produced an interesting report that analysed the cognitive biases of their staff and suggested possible decision-making tactics to mitigate these biases [45,46]. They focused on four decision contexts where biases may occur: confirmation bias (seeking information to confirm existing beliefs), complex decision contexts (increasing the occurrence of biases in logical reasoning), sunk-cost bias (continuing a failing project), and the context and social environment influences on decisions (making assumptions based on personal experience that do not necessarily apply to people in different circumstances) [46]. The examples to overcome these biases were similar to the ones previously described in management, such as deliberate processes to produce counter viewpoints [46,47]. The biases/decision contexts were presumably chosen, as they were well known biases and were used to illustrate approaches to mitigate biases. There was no discernible analysis or rubric for choosing those biases, nor was that the goal of the report. Thus, it was unclear how a governmental department should firstly decide on which biases might be apparent and then, secondly, on how to address those biases. There was no apparent research base on which to choose the best bias mitigation strategy for use within a public administration organisation.

A key challenge when applying specific decision-making rules in a policy environment is its complexity relative to corporate environments, engineering, and medicine [48]. The process of policy decision-making in health promotion is more complex than decision-making in other contexts, where decision-making could take place in a linear and structured process [49]. Policy scholars have developed a number of theories to better conceptualise the complexities of the policy process. These theories focus on different aspects of the policy process and make different assumptions about how public policy is made. They included focusing on institutions (institutional rational choice); ideas and belief systems (the advocacy-coalition framework); interactions between problems; political issues and policy solutions (multiple-streams theory) [50]; and the interrelationships between actions and policy decisions across levels of government [51]. Recent attempts to examine how actors have shaped the policy process included network analysis, social movement theories, and political sociology.

Clavier and de Leeuw view policy change as a multifaceted concept which can occur at different moments in the policy process (i.e., following elections or after a public crisis, such as an epidemic) [49]. It may happen as a response to a change in the balance of power between actors involved in a policy or as a result of the influence of new knowledge. The scope of public policy change could also differ with some changes resulting from a dramatic paradigm shift or, contrastingly, as a result of small steps. Thus, at the broad level of policy change, it would be exceedingly difficult to nominate a specific time and process whereby a formal decision-making process to mitigate biases could occur. Thus, the techniques used to mitigate biases in other industries would need to be tested and potentially adapted for policy-making environments.

There has been, however, work exploring the connection between cognitive biases at the system level of policy development through integrating political science theory [37]. The challenge in developing this type of model is the number of biases that could influence the policy process [52]. Inherent in this challenge is the paradox of needing to make the model simple enough so that it does not overload people’s cognitive capacities and will actually be used, given the complexity of political science theories of the policy process [9]. There has also been work on how to mitigate biases of public administration staff at the individual capacity level [28]. It has also been highlighted how public health administration could utilise emotional regulation models to improve decision-making, and it was hypothetically shown how this could have resulted in better COVID-19 policies [7]. It is unclear, however, whether public health administrators and government departments have been using these types of decision-making models. Both the UK and Australian governments COVID-19 inquiries [11,12] present terrific opportunities to examine how public health policy decisions are made and whether research from cognitive and social psychology and management science is being used. This is a clear example of an opportunity presenting itself for policy reform [49], in this case of how public health decisions are made. This is an urgent research and practice gap, as evidenced by the number of COVID-19 policies that could have been attributed to cognitive biases. We posit that the mitigation of cognitive biases and improvement in decision-making quality could potentially be achieved by building policy capacity at the organisational level through application of this research base. However, we need as a first step to understand in more detail current decision-making processes that are being used (or not) in public health administration.

## 4. The Way Forward: Research-Informed Practice

Organisational psychology research is another area that can provide a practice guide for mitigating cognitive biases with public health administration organisations. The first step is to examine the workplace environment within public health administrative organisations to determine whether this may be exacerbating cognitive biases. Moss et al. showed how a workplace environment that fostered a sense of meaning and purpose in people’s lives could alleviate a number of cognitive biases [53]. Their particular focus was how these biases were related to burnout [53], which had been demonstrated in public administration, as well as in many other workplaces [54]. A workplace environment that could reduce a number of biases simultaneously could also improve the decision-making capability, which could then be related back to the central definition of policy capacity.

A related research agenda is to provide some typology of biases and mitigation strategies that can be applied within a public health administration context. Moss et al. conducted a systematic review of cognitive biases related to workload and burnout and distilled this list down to 14 biases [53]. They grouped these 14 biases into 4 conceptual categories, demonstrating how a workplace environment that provided meaning could address all these biases. A comprehensive process model for evidenced-based decision-making primarily for corporate organisations was based, in part, on thinking about three types of human cognitive biases, grouped as those based on external influences, those based on the internal effects (states of mind), and those based on the perceptions of external events [55]. We argue that a similar process needs to be applied to ascertain a common set of biases and the potential mitigation strategies for public health administration domains. For instance, there may be common biases in different thematic policy domains, such as social policy contexts, infrastructure, taxation policy development, and other areas, or a common model that could address many cognitive biases peculiar to a policy-making environment, akin to Parkhurt’s [37] model or Banaseiwicz’s [55] evidence-based decision-making model for organisations. From this research base, specific decision-making rules and processes could be developed for certain topic areas.

There may be other research being conducted on specific topics that could also assist in developing decision-making rules and processes that could mitigate cognitive biases in public health administration work. Some of the strategies currently recommended for addressing gender bias [42], or the need to address the historical context of racial bias [44], may have pertinence to other topics as well. In addition, it is conceivable that public participation processes could improve administrative decision-making by countering some of these biases, given that group diversity could potentially improve decision-making [14]. Theoretically, a more inclusive process should counter any groupthink or bias evident amongst a small group of professionals. However, the experience of public participation in administration has rarely been that of being influential in the decision-making process [56]. Whether particular participation structures and processes could assist in mitigating biases could be an area for future research.

As documented in this review, there are a number of different types of decision-making strategies and rules, some noted as being more effective than others [8]. There are some decision-making rules or processes that may exacerbate biases rather than reduce them [8,37] and, at this stage, very little guidance whatsoever of what strategies and rules to use in a public health administration decision-making environment. Undertaking this research, as proposed in this review, could make an important contribution to policy capacity research and improve practices in public health administration. It was demonstrated that a particular group structure and process could mitigate cognitive biases in climate change [57], and there have been suggestions that particular decision-making strategies for reducing biases could have assisted with COVID-19 [58].

There have also been recommendations that a more deliberate and reflective decision-making process, involving strategies such as encouraging dissent and viewing alternative framing of the problem, could have resulted in improved decision-making processes during COVID-19 by reducing groupthink (tendency to seek consensus at the expense of greater decision-making deliberation) and other biases, such as confirmation bias [40]. As discussed, it has also been postulated that the use of emotional regulation in decision-making processes could have improved the COVID-19 policies in the U.K. [7]. These strategies are already widely used in other industries, particularly medicine, engineering, and business. The challenge outlined in this paper is finding the opportunities within the public health policy-making process to implement structured decision-making, particularly given the complexity of the process. This opportunity for reforming decision-making could be implemented at the organisational level of governmental departments and in public-sector organisations. Further research is required on the current use of such decision-making models within public health administrations and, if found to be absent, how they can be incorporated. The COVID-19 inquiries, such as those in the U.K. and Australia [11,12], present a timely chance to review these decision-making processes, but it is unclear the extent to which this opportunity will be realised.

## 5. Conclusions

This article highlighted examples of how cognitive biases have hindered effective policy responses to COVID-19, which has been extremely costly from a health and economic perspective [2,4,6,7]. Further research is required to determine the extent to which these biases have impacted COVID-19 policy decision-making. Furthermore, these biases could have potentially been mitigated with appropriate decision-making techniques which are being used in other industries [8]. There is a profound need to understand how to incorporate decision-making processes in order to help mitigate cognitive biases in public health policy-making environments and to address current and future public health policy problems, both pandemic related and more broadly. It is not just COVID-19 but also climate change that have been considered as the most important public health issues of this century [59], and decision-making biases have been evident; a particular group structure and process could mitigate these cognitive biases [57]. The current high profile COVID-19 inquiries provide an opportunity to review the extent to which decision-making biases have been present and then provide recommendations on how to implement the best possible decision-making processes in public health administrations.
